# Annotations

**Published:** 1904-11-26

**Authors:** 


### ANNOTATIONS.

### Wanted, Ten Thousand a Year.

The letter we publish to-day from Mr. Hugh Smith, chairman of the executive committee of the King's Hospital Fund, shows the excellent work done for the London hospitals, on the initiative of H.M. the King, through his Fund. With the end of 1904 the King's Fund will have distributed upwards of half a million pounds, have added to the free hospital beds available for the sick poor about 450 beds, have inspected and fully reported upon every London hospital, and have helped to immeasurably improve the buildings and equipments of the majority of these institutions. Such a record, in so short a time as seven years, should ensure a gift to the Fund, at this season, from all thoughtful Londoners at any rate. Mr. Smith asks for £10,000 a year to increase the permanent income of the Fund by that amount. Most people would be glad of such an income, and for this reason relatively few can have it in their power to respond effectually. It follows, therefore, that the only way to secure such a sum for the King's Fund is to approach directly and personally those who have this power to give. There are connected with the King's Fund many wealthy men, some of whom have apparently not given—largely, at any rate—to the King's Fund. There are men of wealth in the House of Lords, in the City, and elsewhere who should be approached without delay. Ten men might give, for instance, the equivalent of £1,000 a year each, or two might give the equivalent of £5,000 a year each. The great thing is to set to work systematically, and to lay the facts in the right way before those who have the power to respond in sufficient amount to this appeal. We should like to see one-half of the money given by a man like the Duke of Devonshire, for instance, and the other by someone in the City. The raising of money is not an easy matter in times like the present, and any attempt in this direction must be made with intelligence, on a definite plan, to the right people, in the right way. Mr. Smith's effort ought to succeed, and will succeed, we are confident, if the course here recommended be followed energetically.

### Jewish Opposition to a Jewish Hospital.

The offer of a Leeds Alderman to contribute the sum of £4,000 towards the erection of a Jewish nursing home in that city is exciting formidable opposition. Mr. Moser says that he was prompted to make the proposal by a desire to erect a memorial to Dr. Herzl, and in order to comfort the afflicted on the widest possible lines. But there is no question as to the excellence of his motives, and those who demur to the scheme thoroughly appreciate his generosity. One of the objections is that the scheme only provides for the equipment, partial endowment, and maintenance of the institution during its first year; it would, therefore, only continue to exist if it received the full support of the Jewish community. This support is not likely to be forthcoming, because in the Leeds General Infirmary and Dispensary the requirements of the Hebrew population are already sufficiently studied and their religious scruples always respected. Moreover, as the proposed hospital would have but 10 beds, and there are usually many more than 10 sick Jews in Leeds, the others would still have to go to the Infirmary, whilst the money of the richer Jews went to the Home. In that event the Infirmary authorities would hardly be blamed if they failed to admit the surplus patients. Among those who object to the scheme is the President of the Great Synagogue in Belgrave Street, who, claiming to speak on behalf of the five congregations in the city, declares that there is no need for a Jewish hospital because the Jews are as well treated by the authorities at the General Infirmary as the Christians. "Besides," he added, "it would only tend to create racial differences." Here, in our opinion, is an overwhelming reason why the enterprise should be abandoned. It is the pride of our great hospitals that they are absolutely unsectarian, and on that ground they are able to appeal for help to all sorts and conditions of people irrespective of race or religion. So far, Mr. Alderman Moser has declined to entertain the alternative idea of endowing a certain number of beds at the Infirmary, but we hope that he may yet be persuaded to adopt it. We do not forget that a Jewish hospital has just been opened at Manchester, but the multiplication of institutions of this class, wherever the wants of Jews and Christians are alike amply provided for, is to be deprecated instead of encouraged. They must inevitably tend to accentuate differences which should entirely disappear in the care of the suffering.

### Physical Education.

The importance of the culture of physical development as a factor in the promotion of national welfare is not a new discovery. Individual voices have proclaimed it many a time and oft, but they have not been able to attach many followers to their attempted crusade. Even the highest educational authorities have lent their official countenance to the doctrine, and have promoted the practice of something like systematic physical drill in elementary schools. Yet it must be admitted that the great body of the people remain unconcerned and apathetic. Interest, it is true, abounds in forms of exercise which express themselves in the shape of games and sports, and which can be organised into a public spectacle. But the perception of the value of physical training as a national interest of the first order, is not more lively than is the appreciation of the importance of efficient elementary and secondary education. It is this deadness of public opinion which forms the serious obstacle to progress in each of the two directions just indicated. All the stir, agitation, and bustle leave, it is to be feared, the public conscience untouched. Failure to recognise the significance of all this has been responsible for the collapse of more than one promising plan, and it is to be hoped that the active promoters of the latest scheme will not fall into a similar error. The hope for an improvement in the physical well-being of the nation lies much less in elaborate schemes and eloquent speeches than in the prosecution of sound methods in the schoolroom and the playground, and in the cultivation of a public policy which will secure healthy homes and surroundings for the toiling millions of our great cities. When these ends are attained, healthy forms of exercise will follow as a matter of course.

